# Supplementary material for: Configurable topological textures in strain graded ferroelectric nanoplates
Source: Nat Commun. 2018 Jan 26;9:403. doi: 10.1038/s41467-017-02813-5 (PMC5785989; doi:10.1038/s41467-017-02813-5)
Supplement: Supplementary file 1 — Supplementary Information [file 41467_2017_2813_MOESM1_ESM.pdf]

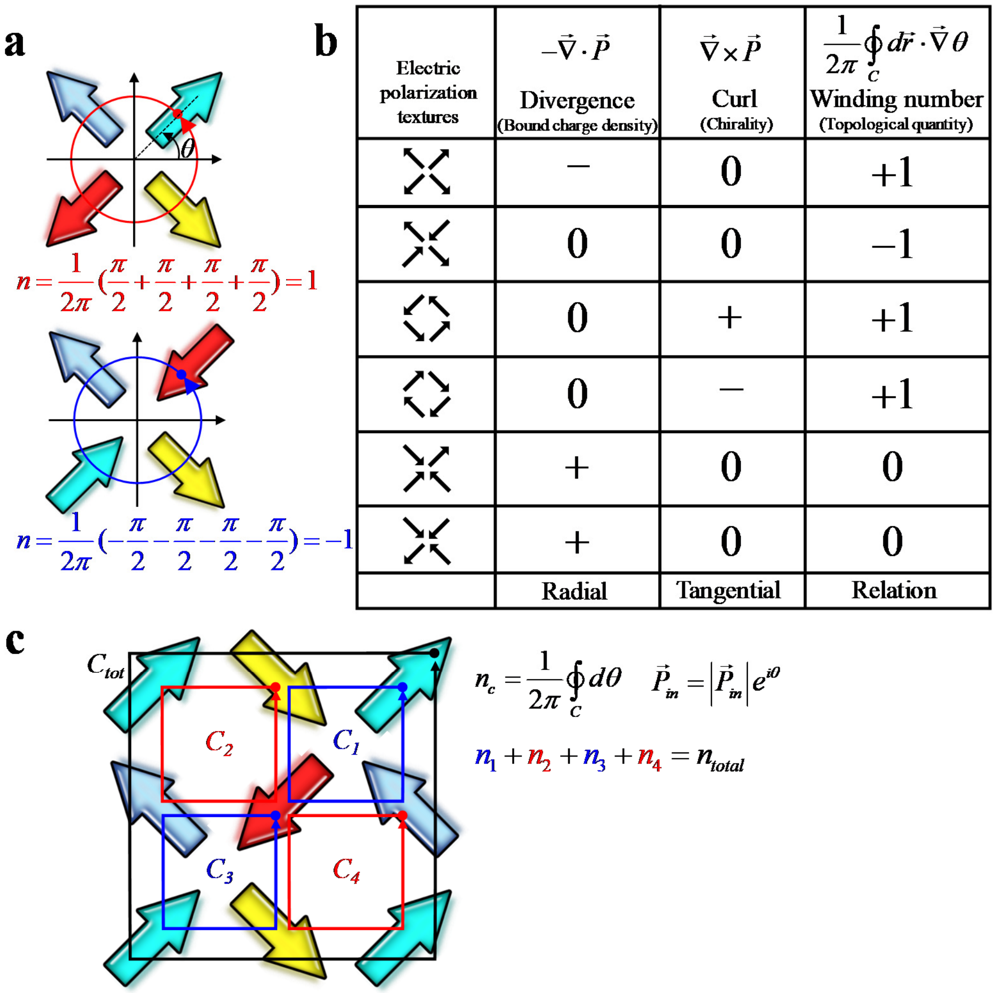

Supplementary Figure 1: The topological properties of the winding number a, Two representative singular points giving the winding number +1 and −1 by the contour integral, respectively. **b**, Various electric textures characterised by physical quantities and the winding number that only takes on integers. **c**, The conserved property of the winding number. The total winding number of an entire system is equal to the total sum of individual winding numbers.

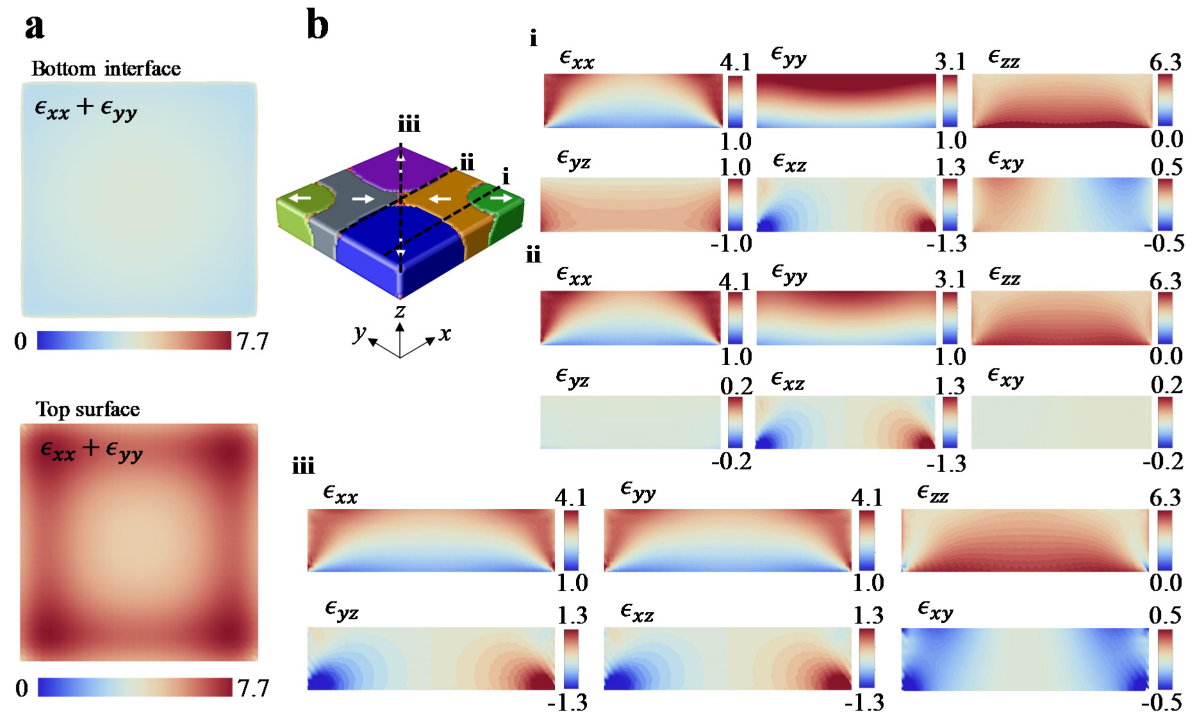

Supplementary Figure 2: Phase field simulation of the strain distribution A three dimensional nanoplate with the bottom interface compressive strained was simulated. For the strain distribution, all the order parameters are maintained as zero, and the strain distribution is caused by the mechanical boundary conditions of the nanoplate. **a**, Strain maps plotted the summation of ε_{xx} and ε_{yy} for the bottom interface and top surface, respectively. The gradient of the normal strain is large at the four corners, which results in upward polarisation domain by strong flexoelectric interaction. **b**, Cross sectional strain maps obtained from the cutting planes i, ii, and iii, respectively. The corner regions at the top were almost relaxed to the bulk lattice parameter (see ε_{xx} and ε_{yy} maps). The inhomogeneous shear strain results in the quadrant domains (see ε_{yz} and ε_{xz} maps). The unit of strain is percent in scale bars.

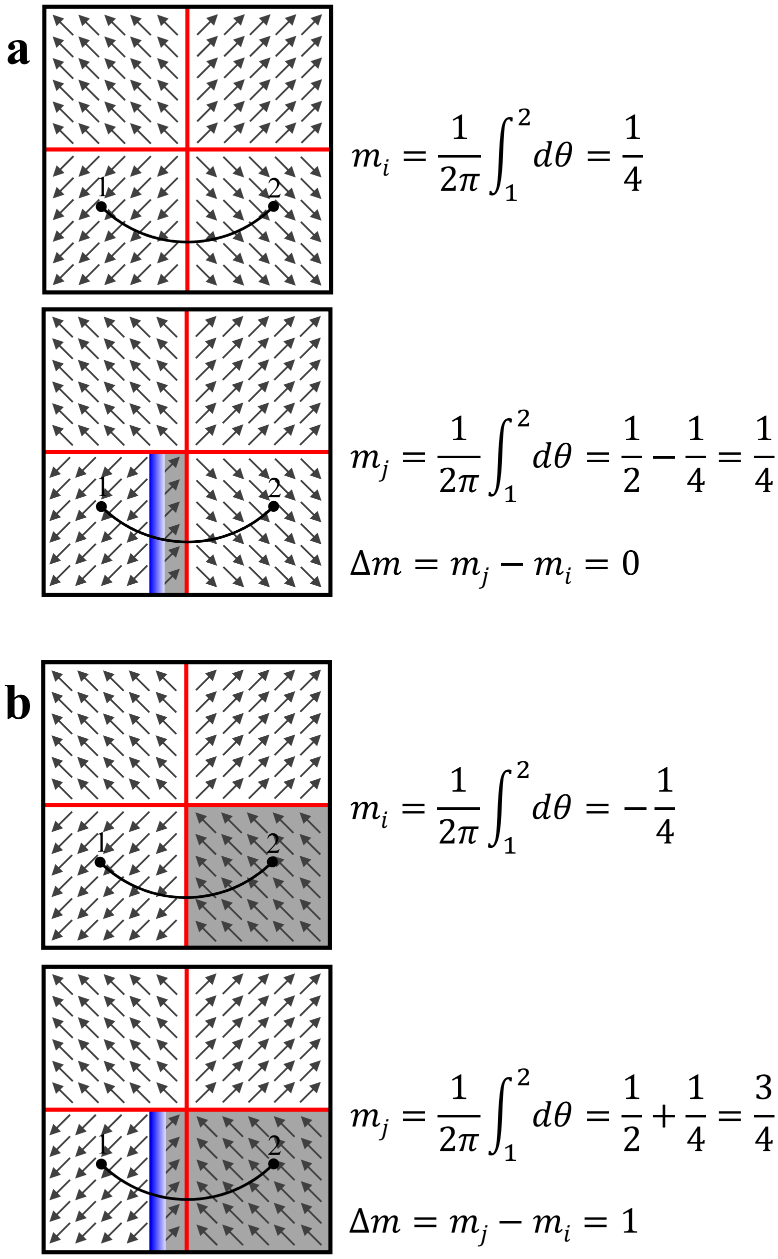

Supplementary Figure 3: The effect of the buffer domain on the winding number calculation The existence or non-existence of a buffer domain can affect the integral value of polarisation angle changes along the path from point 1 to point 2, depending on whether the polarisations of the third and fourth quadrants are outward together (as shown in **a**) or one is outward while the other is inward (as shown in **b**). In the second case, the introduction of a buffer domain increases the integral value by +1.

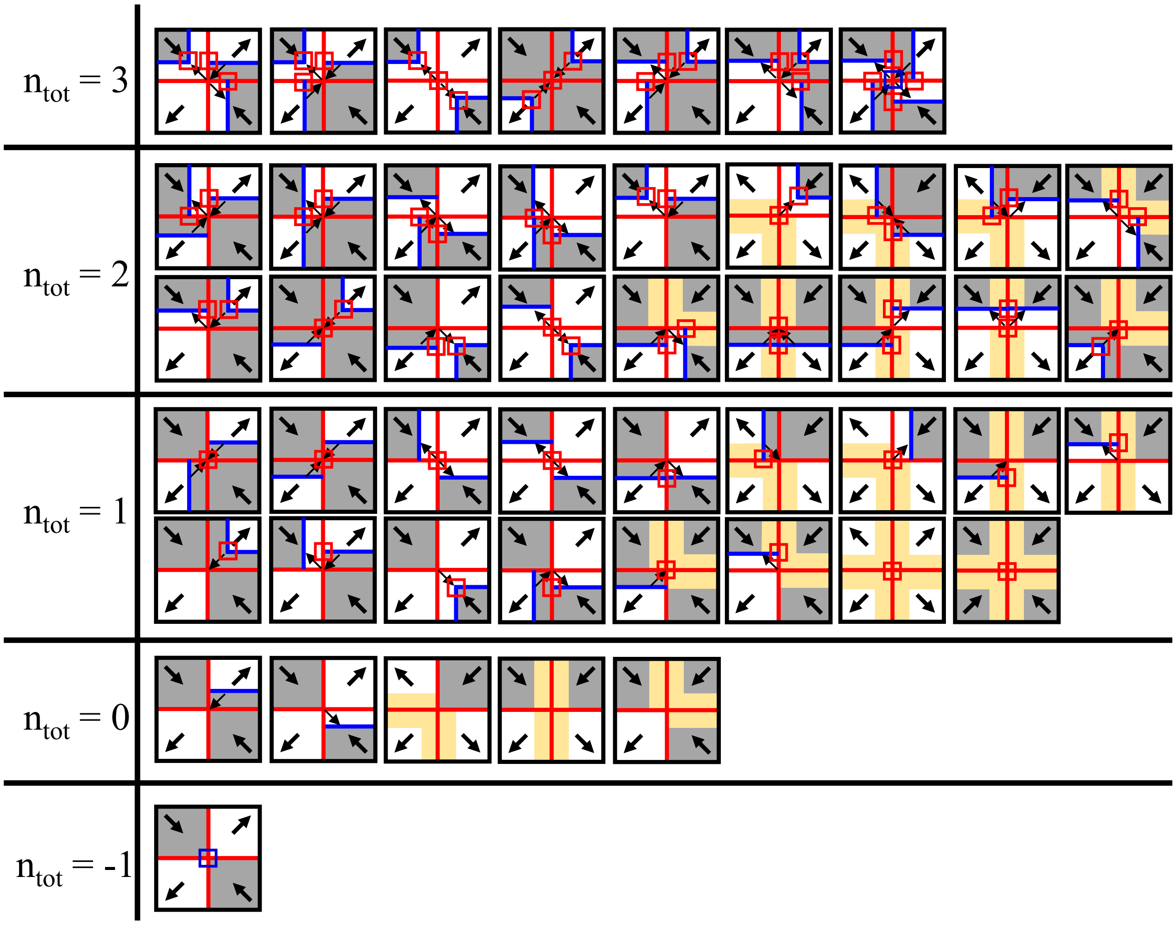

Supplementary Figure 4: List of all possible radial quadrant domain configurations classified according to the total winding number BFO nanoplates can have the total winding number from −1 to 3 according to the switching of quadrant domain(s) and the buffer domain locations. Ferroelastic walls (red lines) surrounded by yellow regions can have a buffer domain on any side or no buffer domain (three cases are allowed) to keep the same total winding number. Each configuration with yellow regions concisely represents multiple variants associated with the arbitrariness of buffer domain location. Furthermore, considering the rotation and mirror symmetry operations (*4m*), the representative domain configurations plotted here can be extended to 1296 ($2^4 \times 3^4$) possible configurations. White (dark) contrast represents OOP polarisation up (down) and blue lines stand for ferroelectric walls.

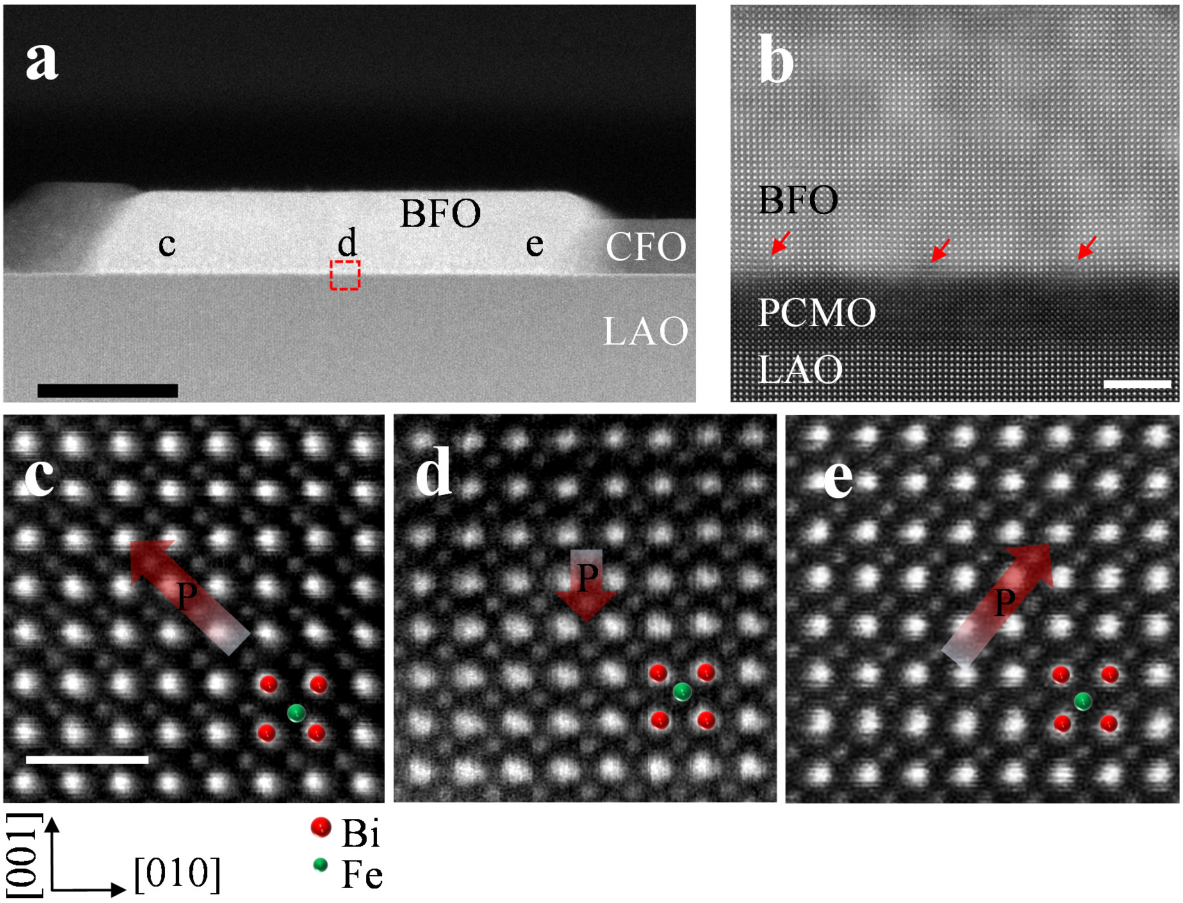

Supplementary Figure 5: TEM characterisation of the electric polarisations of a BFO nanoplate a, Low-magnification dark field STEM image of an overview of a nanoplate. The scale bar represents 100 nm. **b,** HAADF STEM image of the central interfacial region between BFO and LAO (as marked by red dashed box in **a**). PCMO bottom electrode (~4 nm in thickness) is seen to be coherently deposited on LAO substrate. In BFO, periodic misfit dislocations (as indicated by red arrows) are observed along the interface between BFO and PCMO at an interval of ~10 nm. Accordingly, the BFO film in the vicinity of the bottom electrode has an IP lattice parameter of ~3.94 Å as a consequence of the periodic dislocations. Regardless of the strain relaxation, BFO is still under a compressive strain as much as 0.6% at the bottom interface considering the lattice parameter of bulk BFO is 3.965 Å. The scale bar represents 4 nm. **c**-**e**, Atomic scale HAADF STEM images taken from the left, the middle, and the right areas of the BFO plate, respectively. Given that the ferroelectric polarisation is opposite to the off-centre direction of Fe ion accompanying unseen oxygen anions in each unit cell, the left (**c**) and right (**e**) sides of BFO plate exhibit the up-polarisation along [1 -1 1] (or [-1 -1 1]) and [1 1 1] (or [-1 1 1]), respectively. On the other hand, the middle area of BFO plate has a relatively weak down-polarisation. The scale bar represents 1 nm.

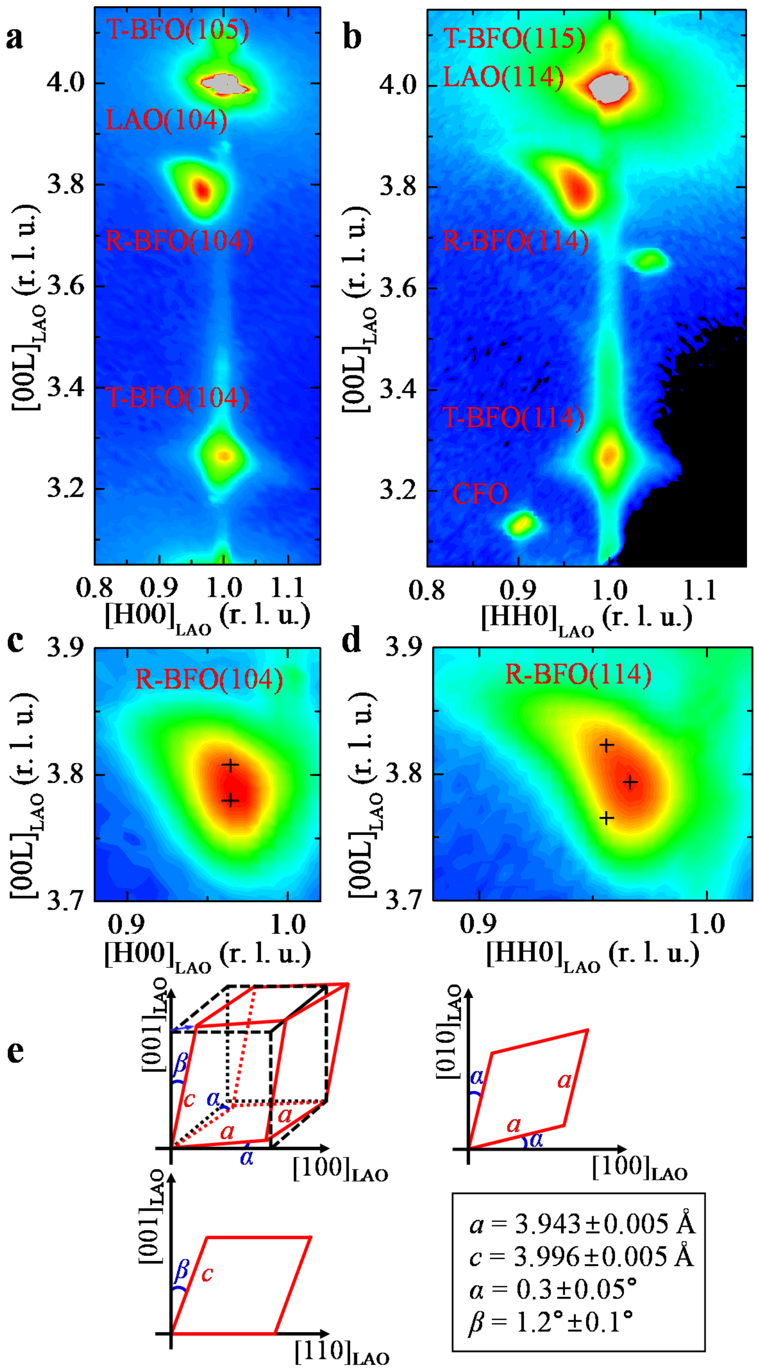

Supplementary Figure 6: Characterisation of the crystal structure by x-ray reciprocal space maps a and **b**, Reciprocal space maps for (104) and (114) reflections at room temperature. The reciprocal lattice unit (r.l.u.) is defined to be $2\pi/3.789$ Å^{-1}. R-BFO peaks deviate from the in-plane reciprocal position of the substrate due to the initial strain relaxation at the bottom layer of BFO nanoplates. **c** and **d**, Enlarged views of the R-BFO peaks. The clear diffuse scattering feature found in the (114) R-BFO peak supports a gradual relaxation of the remaining compressive strain in R-BFO nanoplates. **e**, The crystal structural model of R-BFO unit cell in the first quadrant domain. Based on the model, we can expect the peak positions which are marked by the crosses in **b**.

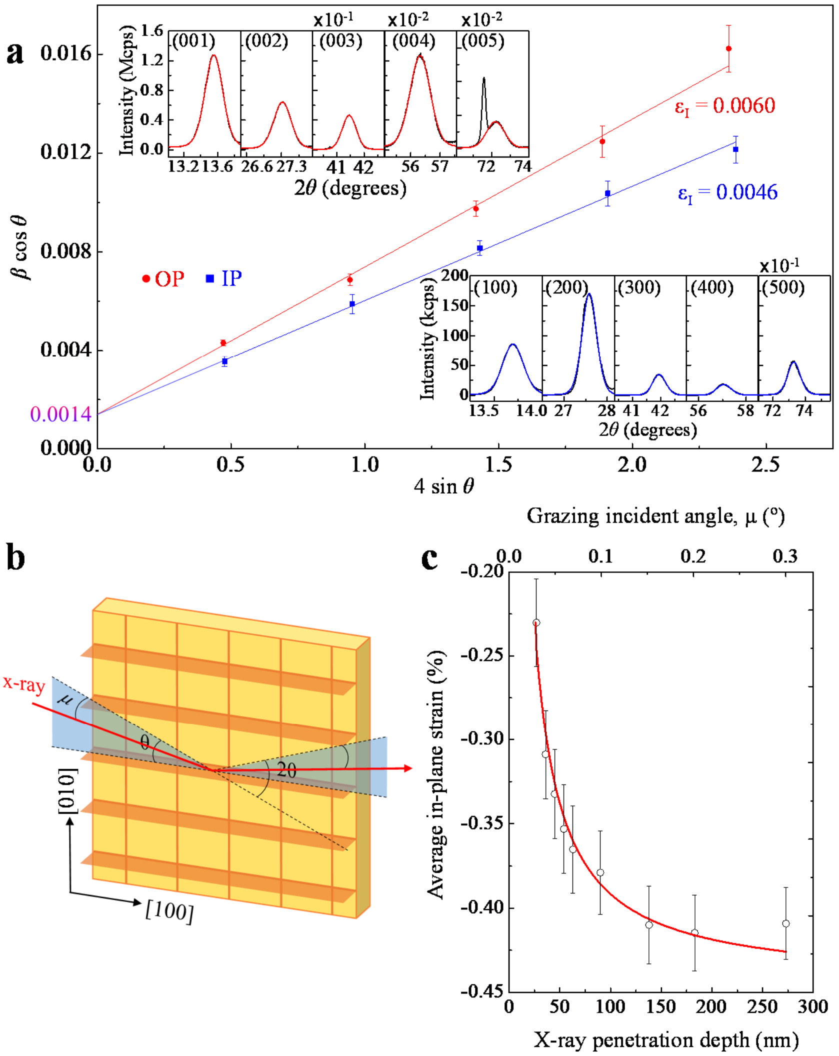

Supplementary Figure 7: Williamson-Hall plots and strain depth profile to evaluate the strain relaxation of BFO nanoplates a, Williamson-Hall plots to obtain the inhomogeneous strains. The θ angle represents half of the 2θ angle of x-ray goniometer at which the scattered x rays are detected. The β indicates the broadness of a diffraction peak detected at 2θ. From the linear slopes, inhomogeneous strain (ε_I), *i.e.* standard deviation of lattice parameter, was estimated to be 0.60% and 0.46% for the OOP and IP axes, respectively. The strain gradient along the OOP axis is estimated to be 0.6% over the film thickness of ~60 nm, *i.e.* ~10^5 m^{-1}. The insets show θ-2θ scans measured for (00L) and (H00) diffraction peaks (black lines) and the peak fitting (Pearson VII) curves (red or blue lines). The error bars are defined by $\Delta\beta\cos\theta + \beta\sin\theta\Delta\theta$, where $\Delta\beta$ is the standard deviation of the peak broadness fitting and $\Delta\theta$ is set to be 0.01°, respectively. **b**, Schematic of the grazing-incidence geometry for determining the depth profile of IP lattice parameters by directly measuring the interspacing of (0 5 0) Bragg planes. The penetration depth of the x ray is in proportion to the grazing incidence angle μ. **c,** Strain depth profile obtained by using the grazing-incidence XRD. The zero strain represents the bulk BFO value 3.965 Å. The red line is the fitting curve based on the strain variation model (see the Methods). The error bars of the lattice parameters are defined as one-tenth of the full-width-at-half-maximum of the diffraction peak.
